# Botox (OnabotulinumtoxinA) for Treatment of Migraine Symptoms: A Systematic Review

**DOI:** 10.1155/2022/3284446

**Published:** 2022-03-31

**Authors:** Negar Shaterian, Negin Shaterian, Aref Ghanaatpisheh, Farnaz Abbasi, Sara Daniali, Maryam Jalali Jahromi, Mohammad Sadegh Sanie, Amir Abdoli

**Affiliations:** ^1^Zoonoses Research Center, Jahrom University of Medical Sciences, Jahrom, Iran; ^2^Student Research Committee, Jahrom University of Medical Sciences, Jahrom, Iran; ^3^USERN Office, Jahrom University of Medical Sciences, Jahrom, Iran; ^4^School of Nursing & Midwifery, Shahid Beheshti University of Medical Sciences, Tehran, Iran; ^5^Research Center for Noncommunicable Diseases, Jahrom University of Medical Sciences, Jahrom, Iran; ^6^Department of Internal Medicine, Jahrom University of Medical Sciences, Jahrom, Iran; ^7^Department of Anesthesia and Intensive Care, Jahrom University of Medical Sciences, Jahrom, Iran

## Abstract

**Background:**

Migraine is one of the most common types of headache, and it is the second most common cause of neurological disorders, with an annual prevalence of about 15% of the population. This study aimed to evaluate the effect of BoNT-A on the duration and intensity of migraine attacks. In addition, we investigated the effective injection sites.

**Methods:**

According to the Preferred Reporting Items for Systematic Review and Meta-Analysis (PRISMA) guidelines, we searched online databases, including Web of Science, PubMed, EMBASE, Scopus, Cochrane Library, ProQuest, ClinicalTrials.gov, and Google Scholar from 2011 to 2021.

**Results:**

A total of 24 articles were included in the study. The use of BoNT-A in individuals suffering from chronic migraine (CM) decreases the frequency of migraine attacks per month, pain intensity, medication use, emergency visits, and migraine-related disabilities. The BoNT-A was well tolerated and leads to improved performance and better quality of life (QoL). Overall, treatment with BoNT-A in adults with CM is beneficial. In addition, the use of BoNT-A in individuals with vestibular migraine (VM) reduces the frequency of migraines and brings about the improvement of disability status caused by migraine headaches. Meanwhile, the use of BoNT-A reduces the frequency of migraine attacks per month among individuals with chronic refractory migraine (CRM).

**Conclusions:**

The use of BoNT-A is a low-cost option for the treatment of various kinds of migraines, including chronic, episodic, unilateral, and vestibular types. BoNT-A can reduce the frequency of migraine attacks per month and diminish the severity of pain.

## 1. Introduction

Migraine is a type of primary headache disorder, and it is the second most common cause of neurological disorders after tension headache with an annual prevalence of about 15% of the population. According to studies conducted in Iran, about 14% of the population was suffering from the disorder in 2016 [1, 2]. Individuals suffering from migraines may have a variety of problems, such as sickness absence and reduced productivity at work, school, and home. Hence, the disease imposes a heavy burden on society [3]. Migraine, as a disease with a genetic background, has a controversial pathogenesis due to the diversity of factors affecting it. In this regard, specific factors such as genes, cytokines (interleukin 1 beta (IL-1B), IL-6, and tumor necrosis factor *α* (TNF*α*)), immune cells (mast cells), and environmental features play an important role. Therefore, there are several treatment options targeting each of the above factors [4–6].

Nowadays, a variety of clinical medications, including *β*-blockers, antiepileptic drugs, calcium antagonists, antidepressants, calcitonin gene-related peptide (CGRP), and onabotulinumtoxinA (BoNT-A), are utilized to prevent migraine [7, 8].

BoNT-A is a complex protein produced by a Gram-positive and anaerobic bacterium called Clostridium botulinum [9]. Initially, it was hypothesized that the mechanism of pain relief by this toxin is due to muscle relaxation and subsequent hypotension [10]. Several mechanisms have been proposed regarding the function of BoNT-A, including inhibiting the exocytosis of neurochemicals and proteins of the motor and sensory systems, reducing exocytosis of proinflammatory cells, neurotransmitters, and excitatory neuropeptides of the nervous system such as substance P, CGRP, glutamate, and inhibiting soluble N-ethylmaleimide-sensitive factor attachment protein receptors (SNARE) [11]. Recent experimental studies have shown that BoNT-A may influence the central nervous system (CNS). The toxin was initially used to treat dystonia and blepharospasm, but following two controlled clinical trials, it was later approved that BoNT-A can also prevent migraine [12]. In these studies, known as the Phase III Research Evaluating Migraine Prophylaxis Therapy (PREEMPT), it was found that BoNT-A injection, compared with placebo, affected the frequency and severity of chronic migraine (CM) and alleviated symptoms. The most common side effects observed after the injection included neck pain, muscle weakness, and pain in the injection site. Repeating this treatment showed that the use of BoNT-A was safe and well tolerated [13].

The practical guidelines for the use of BoNT-A in the treatment of CM, presented by the European Headache Federation (EHF), recommended that 155–195 units should be repeated in the form of intramuscular injection in 31–39 areas around the head and neck at 12-week intervals. Patients should preferably try some other strategies for preventing migraine before starting treatment with BoNT-A [14]. On the other hand, the cost-effectiveness of BoNT-A treatment is an important factor for both the individual and the community. According to the calculations of the National Institute for Health and Care Excellence (NICE), using this method is cost-effective [15]. Since the prevalence of migraine is relatively high and it is associated with disability, this systematic review aimed to evaluate the effect of BoNT-A on migraine attacks. In addition, we investigated the effective injection sites for BoNT-A on migraine attacks.

## 2. Materials and Methods

### 2.1. Search Strategy

The PRISMA (Preferred Reporting Items for Systematic Review and Meta-Analysis) guideline has been used to design the study [16]. The databases of Web of Science, PubMed, EMBASE, Scopus, Cochrane Library, ProQuest, ClinicalTrials.gov, and Google Scholar were systematically searched for relevant studies published between 2011 and the end of 2021. Before 2011, there are some systematic reviews about this topic [17], so we have performed updated systematic reviews. We searched according to MeSH: Migraine (text word) OR Migraines (MeSH term) OR Disorder, Migraine (MeSH term) OR Disorders, Migraine (MeSH term) OR Migraine Disorder (MeSH term) OR Migraine Headache (MeSH term) OR Headache, Migraine (MeSH term) OR Headaches, Migraine (MeSH term) OR Migraine Headaches (MeSH term) AND OnabotulinumtoxinA (text word) OR *Clostridium botulinum* A Toxin (MeSH term) OR Botulinum Toxin A (MeSH term) OR Toxin A, Botulinum (MeSH term) OR Botulinum Neurotoxin A (MeSH term) OR Neurotoxin A, Botulinum (MeSH term) OR Botulinum A Toxin (MeSH term) OR Toxin, Botulinum A (MeSH term) OR Botulinum Toxin Type A (MeSH term) OR Botulinum Neurotoxin Type A (MeSH term) OR *Clostridium Botulinum* Toxin Type A (MeSH term) OR Botox (MeSH term) OR OnabotulinumtoxinA (MeSH term).

### 2.2. Inclusion and Exclusion Criteria

The clinical trial studies conducted between 2011 and 2021 were included in this review without any language restrictions. Nonoriginal articles, including letters, comments, observational studies, and case reports, were excluded. If the language used in an article was other than English or Persian, we asked a translator to translate the article.

### 2.3. Types of Participants

The studies were selected if their participants were people with acute migraine, people 12 years old or older, and people with no systemic disorders.

### 2.4. Types of Interventions

The studies were reviewed if they used BoNT-A for migraine treatment.

### 2.5. Types of Outcome Measures

All included studies were on the number of days with migraine headaches.

### 2.6. Data Extraction

Eligible articles were initially screened by title and abstract. All selected articles were imported into the EndNote X8 software (Thomson Reuters, New York, USA), and duplicated articles were checked and then removed. After removing duplicates, the full text of the qualified records was retrieved and two expert investigators (two first authors, NSH and NSH) evaluated the eligibility of the articles. Afterward, an investigator (AG) extracted the required information, and two others (FA and SD) rechecked them; any discrepancy of opinion or disagreement was resolved by consensus and discussion with the lead investigator (AA) [18]. Data are collected in Table 1.

## 3. Results

**Figure 1:**
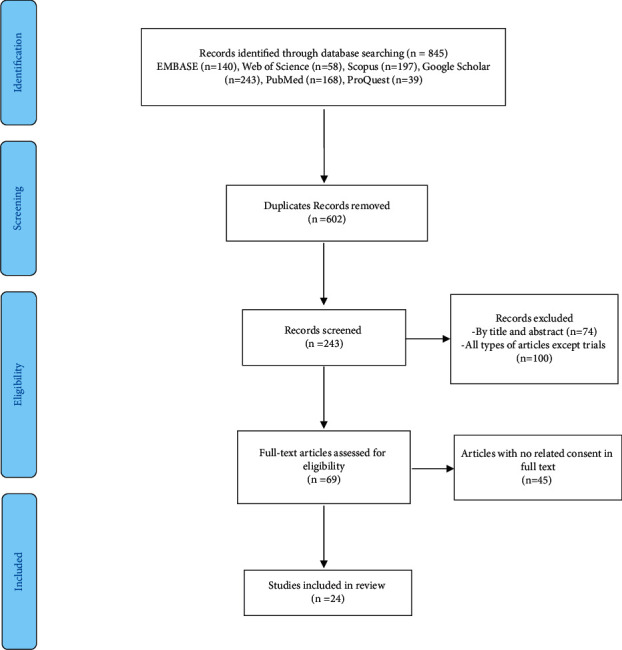
The PRISMA flowchart of the study selection process.

Our search strategy resulted in the retrieval of a total of 845 articles, of which 24 studies were included in the study. In the first phase, 602 duplicate articles were excluded from the study. Subsequently, 174 nonclinical trial studies and unrelated studies according to the title and abstracts of articles were excluded from the study. Next, the full text of 69 articles was reviewed and 45 articles were excluded. Finally, 24 high-quality articles evaluating the effect of BoNT-A in the treatment of migraine from 2011 to the end of 2021 were included (Figure 1 and Table 1). Figure 1 shows the article selection process according to the PRISMA protocol.

The countries in which the articles had been published were as follows: the United States (*n* = 9), Turkey (*n* = 3), the United Kingdom (*n* = 3), Spain (*n* = 3), Germany (*n* = 2), Italy (*n* = 2), Brazil (*n* = 2), Canada (*n* = 2), Russia (*n* = 1), Iraq (*n* = 1), Greece (*n* = 1), China (*n* = 1), North America (*n* = 1), Europe (*n* = 1), Thailand (*n* = 1), Switzerland (*n* = 1), Norway (*n* = 1), Sweden (*n* = 1), and Croatia (*n* = 1).

Most (79.16%) studies evaluated the effect of BoNT-A on CM [19–36]. One study examined the effect of BoNT-A on vestibular migraine (VM) [37], one study evaluated chronic refractory migraine (CRM) [38], and one study examined chronic and episodic migraine (EM) [39]. Moreover, one study did not mention the type of migraine [40]. The minimum and maximum ages of the participants were 12 and 86 years, respectively. Additionally, the lowest and highest numbers of migraine attacks per month were reported as 11 and 20 days, respectively. The doses of Botox used varied in different studies (from 2.5 to 200 units), but most studies (58.3%) used 155 units of Botox. Indeed, one study did not mention the dosage [31].

In the 15 articles of PREEMPT [19–29, 31, 34, 35, 37], the sites of BoNT-A injection were as follows: two sites in the corrugator, one site in the procerus, six sites in the occipital, eight sites in the temporalis, four sites in the frontalis, six sites in the trapezium, and four sites in the cervical paraspinals. In other articles [30, 33, 36, 38–40], the sites of BoNT-A injection were different, which included the forehead, the back of the head, temples, upper back, behind the shoulder blades, neck, nose bridge, bilaterally to frontal muscles, temporal muscles, occipital muscles, semispinalis capitis, splenius capitis, trapezius muscles in the cervical region, occipitofrontalis corrugator supercilii, and acupoint sites.

According to 11 studies [20–24, 26–29, 31, 32, 34, 36], BoNT-A reduced the frequency of CM among patients. Meanwhile, one study found that the same is true for CRM. In addition, three studies [24, 34, 36] showed that BoNT-A reduced pain intensity in patients suffering from CM. Furthermore, one study [30] demonstrated that the use of BoNT-A had no significant effect on CM. However, three studies [19, 25, 33] claimed that BoNT-A is well tolerated in patients with CM. Findings from two studies [22, 29] showed that the use of BoNT-A in CM treatment led to reduced use of other drugs. In addition, one study [29] found that using BoNT-A reduced emergency visits. Findings from two studies [24, 34] showed that treatment with BoNT-A in individuals with CM improves health-related quality of life (QoL). In addition, one study [37] found that BoNT-A reduced the incidence of VM and improved the function; this was also reported in a study on CM. Meanwhile, another study proved that the use of BoNT-A was generally effective in adults suffering from CM. Three studies used BoNT-A for the pediatric and adolescent population [19, 41, 42], in which treatment resulted in reduced frequency of headache, duration of migraine, and pain intensity.

## 4. Discussion

The results of this study showed that Botox is an option with high potential for treating migraine as an inflammatory neurological disease. However, taking advantage of BoNT-A in individuals suffering from CM reduces the frequency of migraine attacks per month, the severity of pain, use of other drugs, emergency visits, and disabilities associated with migraine headaches. Furthermore, BoNT-A is well tolerated and leads to improved performance and health-related QoL. Generally, treatment with BoNT-A in adults with CM is beneficial. In addition, the use of BoNT-A in individuals with VM reduces the frequency of migraine attacks per month and improves the disability status caused by migraine headaches. Moreover, the use of BoNT-A decreases the frequency of migraine attacks per month among those suffering from CRM. Apart from Botox, there are other treatment options. However, considering the availability and cost-effectiveness, Botox is assumed a novel and cost-effective option for these patients.

The effectiveness of BoNT-A has been studied from various perspectives and by different groups [43–45]. Mimeh et al. evaluated the safety and efficacy of BoNT-A toxin in the prophylactic treatment of adults with CM compared with placebo. They reported that BoNT-A treatment is well tolerated in adults with CM, and it can be considered as a safe method in this regard. However, BoNT-A is associated with an increased risk of side effects, and there is still uncertainty about the effectiveness of BoNT-A in comparison with placebo [46]. A systematic review and meta-analysis by Herd et al. evaluated the effect of botulinum toxin on migraine prevention among adults, and the results showed that botulinum toxin treatment alleviated the severity of CM and EM. Besides, in CM, botulinum toxin reduced the migraine frequency by two days a month and had an acceptable safety profile. However, excessive use of migraine medications does not prevent the effectiveness of botulinum toxin. There is also no evidence to support or rule out the effectiveness of the toxin in EM. In addition, the relative risk of side effects associated with botulinum toxin treatment was measured as twice higher than placebo; however, considering comparison standards, it had a lower risk and withdrawal rate (3%) [47]. Another systematic review by Shen et al. investigated the effect of botulinum toxin A on the prevention of adult migraine disorders. By analyzing CM and EM, it was proved that BoNT-A did not decrease the frequency of migraine attacks per month compared with placebo. In addition, the analysis of the migraine disability assessment questionnaire reported by the patients showed that BoNT-A led to a significant improvement. Besides, this study showed that BoNT-A, as a treatment regimen, cured CM after 16 weeks of treatment, although this was not the case for EM [48].

In line with our study, Loeb et al. conducted a randomized controlled trial (RCT) to evaluate CM patients treated with botulinum toxin A (BT-A). After comparing BT-A with low-level laser therapy (LLLT), it was shown that both treatments reduced the frequency of headaches, acute drug use, and pain intensity. Anxiety decreased in the BT-A group, while sleep disorders were lessened in the LLLT group. The results showed that both treatments could be used to treat CM without significant differences [49]. A study by Cheng and Ahmed showed that BoNT-A is a very good option for migraine prophylaxis in adults. In addition, it could be more effective when used in combination with other oral medications, and the low side effects of Botox could be controlled by adjusting the duration of treatment [50]. Moreover, in a prospective, real-life analysis by Ahmed et al. positive data from long-term treatment and follow-up of at least two years of patients with CM showed that they still appropriately responded even after two years. Additionally, it has been observed that CM subsequently changed into EM in some patients [51]. A systematic review by Argyriou et al. showed some conflicting results about the effectiveness of BoNT-A in primary headaches (PHs) other than CM. However, BoNTA may be a treatment option for patients who do not respond to common migraine prophylaxis. Based on the available limited evidence, BoNTA may be used for refractory tension headaches, trigeminal autonomic cephalalgia, primary headaches, nummular headache, hypnic headache, and daily persistent headache. In this regard, the initial nature of cephalalgia and other unsuccessful medications should be considered. Therefore, experienced BoNTA therapy physicians are required to guide the treatment protocol for each patient to optimize good and safe outcomes [52].

Consistent with our study, in a clinical trial, Naderinabi et al. randomly divided 150 eligible patients into different groups of receiving acupuncture (A), botulinum toxin A (B), and control (C). During the 3-month study, pain intensity significantly decreased in all three groups, with a further decrease in group A. Frequency of migraine attacks per month, sickness absence, and the need for medication were evaluated three times among the participants of all groups. It was proved that all mentioned problems were significantly decreased and fewer side effects were seen in group A [53].

A meta-analysis by Affatato et al. [54] evaluated the effectiveness of onabotulinumtoxinA treatment in migraine patients with depression. They included eight studies for meta-analysis. Consequently, it was shown that onabotulinumtoxinA treatment significantly reduced the severity of CM and major depressive disorder in patients simultaneously suffering from both. Comparative analysis showed a strong equivalent effect in monomorbid and comorbid patients, with beneficial and specialized effects for some migraine characteristics [54]. Migraine during pregnancy is a widespread issue. According to some studies, migraine during pregnancy can increase the risk of maternal stroke and hypertensive disorders [55]. Therefore, finding a safe treatment option for this group of patients is essential. In a study by Wong et al., the effect of BoNT-A on 45 pregnant patients with CM was evaluated. Although the sample size of the study was small, the findings showed that BoNT-A had no adverse effects on pregnancy and birth [56]. However, children born from mothers diagnosed with migraine and treated with BoNT-A need to be followed up.

Schoenbrunner et al. [57] compared the cost-effectiveness of long-term botulinum toxin type A with surgical deactivation of trigger sites for the treatment of migraine headaches. In this study, the Markov model was developed, and the costs, utility, and other inputs of the model were determined from other studies. According to this model, surgical deactivation of trigger sites is a more cost-effective option for refractory migraine headaches. The model showed that surgical deactivation of trigger sites is more effective and less costly than long-term and targeted botulinum toxin type A over a patient's lifetime [57]. This study is inconsistent with our findings because this study showed that due to the reduction in the use of other medications and emergency visits, treatment costs are reduced, and hence, this method is cost-effective. On the other hand, since Schoenbrunner et al. [57] compared the cost of treatment with another type of treatment (deactivation) that not examined in our study, this inconsistency is justifiable. The results of this study will help clinicians to enhance their knowledge about which type of migraine could be treated by Botox, which is the best place for Botox injection, and what is the appropriate dose for each type of migraine.

Previous systematic reviews performed their analysis on adults [47, 48], but we did not have an age restriction. In this regard, three studies used BoNT-A for the pediatric and adolescent populations [19, 41, 42]. A study conducted by Shah et al. on children with migraine showed that patients with CM aged between 8 and 17 years who received BoNT-A experienced lower frequency and severity of migraine attacks than those receiving placebo [42]. On the other hand, in a 5-year retrospective longitudinal study, Shah et al. examined pediatric patients who were treated with BoNT-A because of their CM pain in an outpatient clinic [41]. After treatment, it was observed that the frequency of headaches, duration of migraine, and pain intensity were reduced. Besides, no serious side effects were reported [41]. Winner et al. [19] used Botox for the prevention of headaches in adolescents (12 to <18 years) with chronic migraine. They found that Botox was well tolerated in adolescents, and the treatment led to a reduced frequency of severe headache days [19].

### 4.1. Strengths and Limitations of This Study

One of the strengths of our work is the precise descriptive table that inserted the details of each included study. Furthermore, we did not have an age restriction for including the study.

Similar to other studies, our research had some limitations. There is a significant level of heterogeneity among studies for patients' age, sample size, location, type of migraine, duration of migraine (days/month), Botox doses, unit of Botox dose, and site of Botox injection. Hence, due to the high heterogenicity among the studies, we could not perform a meta-analysis. Lack of online registration (PROSPERO) was another limitation to this study, because the data were already extracted (we pre-extracted the data and were unable to register it according to PROSPERO). However, there were no similar studies in the PROSPERO database since the search was performed.

## 5. Conclusions

In general, it can be noted that the use of BoNT-A, which is a type of Botox to treat migraines, is an effective and cost-effective option for the treatment of migraine. On the one hand, migraine disrupts QoL and can cause disability. BoNT-A treatment has effective benefits such as reducing the frequency and severity of headaches, improving disability status, and positively influencing health-related QoL. Furthermore, BoNT-A treatment reduces the use of other medications and emergency visits, both of which make BoNT-A treatment more cost-effective. No side effects other than neck pain have been reported following BoNT-A injection for several days. Further studies are recommended to determine whether treatment with BoNT-A has long-term side effects and whether it can be used as a treatment option if there are some underlying diseases. In addition, future studies may investigate the safety of using BoNT-A in pregnancy without causing complications for the mother and fetus.

## Figures and Tables

**Table 1:** Overview of all included studies in the systematic review.

No	Reference	Study type	Location	Sample size	Age		Migraine days/month		Migraine type	Botox dose (unit)		The site of Botox injection	The outcome of using Botox
1	Görür et al. [37]	CT1	Turkey	60	45.86		NR2		Vestibular	155		Corrugator 10 units/ 2 points; procerus 5 units/1 point; frontalis 20 units/4 points; temporalis 40 units/8 points; occipital 30 units/6 points; cervical paraspinalis 20 units/4 points; trapezium 30 units/6 points, (PREEMPT^6^)	Decreased attack frequency in vestibular migraine patients and improvement in the migraine disability assessment score

2	Alshalah et al. [40]	CT	Iraq	100	NR		20		NR	155-195		7 specific muscles in the head and neck in 6 zones: 1. forehead. 2. The back of the head. 3. Temples. 4. The upper back, behind the shoulder blades. 5. Neck. 6. Nose bridge	Decrease in headache-day frequency

3	Akçay [36]	CT	Turkey	53	Group 1	68	≥ 15		Chronic	Group 1a	5	Bilateral to frontal muscles, temporal muscles, occipital muscles, semispinalis capitis, splenius capitis, and trapezius muscles in the cervical region	Decreasing headache-day frequency and the severity of pain treatment in both young and old population
										Group1b	2.5		
					Group 2	37				Group 2a	5		
										Group 2b	2.5		

4	Ahmed et al. [20]	CT	Germany, Italy, Norway, Russia, Sweden, Spain, and UK	641	45.4		20.6		Chronic	155		Corrugator 10 units/ 2 points; procerus 5 units/1 point; frontalis 20 units/4 points; temporalis 40 units/8 points; occipital 30 units/6 points; cervical paraspinalis 20 units/4 points; trapezium 30 units/6 points, (PREEMPT)	Reduction in headache-day frequency
5	Tang et al. [21]	RCT	Brazil	18	42		28		Chronic	155		Corrugator 10 units/ 2 points; procerus 5 units/1 point; frontalis 20 units/4 points; temporalis 40 units/8 points; occipital 30 units/6 points; cervical paraspinalis 20 units/4 points; trapezium 30 units/6 points, (PREEMPT)	A sharp decrease during the treatment and post-treatment phases, compared with baseline

6	Grazzi [22]	CT	Italy	50	51.2		25.3		Chronic	155		Corrugator 10 units/ 2 points; procerus 5 units/1 point; frontalis 20 units/4 points; temporalis 40 units/8 points; occipital 30 units/6 points; cervical paraspinalis 20 units/4 points; trapezium 30 units/6 points, (PREEMPT)	Significant decrease in headache-day frequency and medication intake per month

7	Demiryurek et al. [23]	CT	Turkey	60	34.73		18.78		Chronic	155		Corrugator 10 units/ 2 points; procerus 5 units/1 point; frontalis 20 units/4 points; temporalis 40 units/8 points; occipital 30 units/6 points; cervical paraspinalis 20 units/4 points; trapezium 30 units/6 points, (PREEMPT)	Decreased headache days in 88.3 % of patients

8	Lipton et al. [24]	RCT	USA	1236	41		O/O (onabotulinumtoxinA/onabotulinumtoxinA )	19.9	Chronic	155		Corrugator 10 units/ 2 points; procerus 5 units/1 point; frontalis 20 units/4 points; temporalis 40 units/8 points; occipital 30 units/6 points; cervical paraspinalis 20 units/4 points; trapezium 30 units/6 points, (PREEMPT)	-Significant reduction in Headache Impact Test (HIT-6) and Migraine-Specific Quality of Life Questionnaire (MSQ) for onabotulinumtoxinA versus placebo, in the double-blind (DB) phase -Significant reduction in HIT-6 for onabotulinumtoxinA/onabotulinumtoxinA (O/O) versus placebo/onabotulinumtoxinA (P/O), in the open-label (OL) phase -Improvement in health-related quality of life (HRQoL) in three MSQ domains
							P/O (placebo/onabotulinumtoxinA)	19.8					

9	Vikelis [25]	CT	Greece	119	21-75		NR		Chronic	155 and 40 for additional dose		Corrugator 10 units/ 2 points; procerus 5 units/1 point; frontalis 20 units/4 points; temporalis 40 units/8 points; occipital 30 units/6 points; cervical paraspinalis 20 units/4 points; trapezium 30 units/6 points, (PREEMPT)	The use of onabotulinumtoxinA for the prophylaxis of chronic migraine, as this intervention proved effective, safe, and well tolerated

10	Ahmed et al. [26]	CT	UK	465	47.5		≥15		Chronic	155		Corrugator 10 units/ 2 points; procerus 5 units/1 point; frontalis 20 units/4 points; temporalis 40 units/8 points; occipital 30 units/6 points; cervical paraspinalis 20 units/4 points; trapezium 30 units/6 points, (PREEMPT)	Reduction in headache-day frequency
11	Boudreau et al. [27]	CT	Canada and USA	32	≥ 18		16.8 (*p* < 0.0001)		Chronic	155		Corrugator 10 units/ 2 points; procerus 5 units/1 point; frontalis 20 units/4 points; temporalis 40 units/8 points; occipital 30 units/6 points; cervical paraspinalis 20 units/4 points; trapezium 30 units/6 points, (PREEMPT)	Reductions in headache-day frequency

12	Hou, M [39]	RCT	China	102	>18		NR		Chronic and episodic	-2.5 U each site -25 U per subject		Fixed sites (*n* = 41) including occipitofrontalis, corrugator supercilii, temporalis, and trapeziue, or at acupoint sites (*n* = 42) including Yintang (EX-HN3), Taiyang (EX-HN5), Baihui (GV20), Shuaigu (GB8), Fengchi (GB20) and Tianzhu (BL10)	Because of more efficient improvement for reducing migraine frequency, density, duration, and associated symptoms in acupoint sites group than in fixed sites groups, the efficacious site for migraine treatment is acupoint sites

13	Cady et al. [28]	RCT	USA	20	48.5		NR		Chronic	155		Corrugator 10 units/ 2 points; procerus 5 units/1 point; frontalis 20 units/4 points; temporalis 40 units/8 points; occipital 30 units/6 points; cervical paraspinalis 20 units/4 points; trapezium 30 units/6 points, (PREEMPT)	≥50% reduction in headache-day frequency
14	Cernuda-Morollón et al. [29]	CT	Spain	132	46.3		NR		Chronic	155-195		Corrugator 10 units/ 2 points; procerus 5 units/1 point; frontalis 20 units/4 points; temporalis 40 units/8 points; occipital 30 units/6 points; cervical paraspinalis 20 units/4 points; trapezium 30 units/6 points, (PREEMPT)	Decrease in consumption of any acute medication and emergency visits (long-term response to onabotA)

15	Hollanda [30]	RCT	Brazil	58	18-85		NR		Chronic	100		Frontal 3U/ 2-4 points, temporal 3U/ 2-4 points, occipital 3U/ 2-4 points	No significant differences between active intervention or placebo groups

16	Aurora et al. [31]	RCT	North America and Europe	1384	18-65		≥15		Chronic	NR		Corrugator 10 units/ 2 points; procerus 5 units/1 point; frontalis 20 units/4 points; temporalis 40 units/8 points; occipital 30 units/6 points; cervical paraspinalis 20 units/4 points; trapezium 30 units/6 points, (PREEMPT)	Significantly reduce headache-related disability and improve functioning

17	Cady et al. [32]	RCT	USA	59	36.9		11.9		Chronic	Up to 200		NR	At least a 50% reduction in headache-day frequency in subjects with chronic migraine

18	Chankrachang et al. [33]	RCT	Thailand	128	18-65		NR		Chronic	120 240		2 subcutaneous injections into both the frontal and temporal regions of the face, and 2 intramuscular injections into the occipital region	Efficacy and tolerability of Dysport as a migraine treatment in particularly 240 U dose
19	Lipton [34]	RCT	USA	1384	41.3		NR		Chronic	155		corrugator 10 units/ 2 points; procerus 5 units/1 point; frontalis 20 units/4 points; temporalis 40 units/8 points; occipital 30 units/6 points; cervical paraspinalis 20 units/4 points; trapezium 30 units/6 points, (PREEMPT)	Meaningful reductions in headache impact and improvements in health-related quality of life (HRQoL(

20	Oterino [38]	CT	Spain	35	24-68		24.7		Chronic refractory	First: 100 U Second: 200 U		One site into corrugator, two into frontalis, three into temporalis, two into suboccipitalis, one into semispinalis and one into splenius/ 5 units per site	-Decrease in headache days per month-18% of patients experienced adverse effects

21	Aurora et al. [35]	RCT	Canada, Croatia, Germany, Switzerland, UK, and USA	1384	18-65		≥ 15		Chronic	155		Corrugator 10 units/ 2 points; procerus 5 units/1 point; frontalis 20 units/4 points; temporalis 40 units/8 points; occipital 30 units/6 points; cervical paraspinalis 20 units/4 points; trapezium 30 units/6 points, (PREEMPT)	Safety and efficacy in adults with chronic migraine

22	Winner et al. [19]	RCT3	USA	115	12 to <18 years		≥15		Chronic	155, 74		Corrugator 10 units/ 2 points; procerus 5 units/1 point; frontalis 20 units/4 points; temporalis 40 units/8 points; occipital 30 units/6 points; cervical paraspinalis 20 units/4 points; trapezium 30 units/6 points, (PREEMPT)4	-OnabotulinumtoxinA was well tolerated in this adolescent population.-In adults, the beneficial effects of onabotulinumtoxinA have been shown to increase with multiple treatments.-The most reported adverse events were neck pain, upper respiratory tract infection, migraine, and nasopharyngitis
23	Shah et al. [41]	RCT	USA	10	8-17		15.5 to 4 days/month		Chronic	A median dose of 165 total units was used per treatment		Corrugator 10-15 units; procerus 5-10 units/; frontalis 10-30 units/; temporalis 40-50 units/; occipital 20-50 units/; cervical paraspinalis 20-40 units/; trapezium 20-40 units	Median pretreatment to post-treatment headache frequency was 15.5 to 4 days/month (*p* < 0.0001), durations were 8 [0, 24] to 1 [0, 7] hours (*p* < 0.025), and intensity was 6 [4, 8] to 4 [2, 5] (*p* < 0.0063). Patients had no serious adverse events

24	Shah et al. [42]	RCT	USA	15	8 to 17 years old		Chronic migraines (at least 6 months), and 15 or more headache days in a 4-week baseline period		Chronic	155 units at 31 injection sites—in 3-month intervals and follow-up visits every 6 weeks			-Treatment resulted in a statistically significant decrease from the baseline values compared with placebo 6-week post-treatment compared with placebo: frequency (20 (7 to 17) vs 28 (23 to 28); *p* = 0.038), intensity (5 (3 to 7) vs 7 (5 to 9); *p* = 0.047), and PedMIDAS (Pediatric Migraine Disability Score) (3 (2 to 4) vs 4 (4 to 4); *p* = 0.047). -There was no statistically significant difference in the duration (10 (2 to 24) vs 24 (4 to 24); *p* = 0.148) of migraines between the two groups

CT: controlled trial, NR: not reported, RCT: randomized controlled trial, PREEMPT: Phase III Research Evaluating Migraine Prophylaxis Therapy.

## Data Availability

All the data are available from the corresponding author upon reasonable request.

## Supplementary Materials

Supplementary Materials The PRISMA checklist for reporting systematic reviews.
